# Successful Collagen Crosslinking in a Patient With Keratoconus and Systemic Scleroderma

**DOI:** 10.7759/cureus.59431

**Published:** 2024-05-01

**Authors:** Ayman Alghamdi, Essam A Alghamdi, Abdullah Alghamdi, Mohammed M Abusayf, Muhammad A Ahad

**Affiliations:** 1 Oculoplastic Division, King Khaled Eye Specialist Hospital, Riyadh, SAU; 2 College of Medicine, King Abdulaziz University, Jeddah, SAU; 3 Community Medicine, King Abdulaziz University Faculty of Medicine, Jeddah, SAU; 4 Ophthalmology Department, King Saud University, Riyadh, SAU; 5 Cornea and External Disease Division, King Khaled Eye Specialist Hospital, Riyadh, SAU

**Keywords:** contact lens wear, keratoconus progression, connective tissue disorder, case report, keratoconus, corneal ectasia, collagen vascular disease, collagen cross linking

## Abstract

Scleroderma is an autoimmune disease that affects connective tissue. Keratoconus (KC) is a rare ocular condition that may appear alongside scleroderma. Contact lenses are an essential visual aid for KC patients, especially in advanced cases. However, scleroderma patients may face difficulties using them due to finger-related disabilities. Corneal collagen cross-linking (CXL) is a crucial treatment used to prevent corneal thinning and visual deterioration in progressive KC. However, the potential trigger of corneal melt and delayed healing following CXL in KC patients with scleroderma is a matter of concern. We present a case of a patient with KC and scleroderma who underwent CXL without any complications.

## Introduction

Keratoconus (KC) is characterized by a conical shape with thinning of the cornea, which results in astigmatism and significant visual loss in severe cases [1,2]. The clinical onset of the condition often occurs during adolescence and continues to progress to varying degrees, with no gender or ethnicity predominance. The exact etiology and pathogenesis of the condition are still not yet widely understood, but eye rubbing and contact lenses were shown to have a significant impact on either causing or aggravating KC [2]. Several new therapeutic approaches have emerged since late 1990 [3,4]. One technique that has grown widely in current practice is corneal collagen cross-linking (CXL). What makes this procedure distinctive is its ability to revolutionize the conventional approach of not only stopping KC progression but also potentially enhancing the corneal tissue while being minimally invasive [5]. CXL uses riboflavin as a photosensitizer, which, when exposed to ultraviolet light long enough, provokes chemical reactions in the corneal stroma, leading to the formation of new bonds between the collagen molecules. This collagen crosslinking strengthens and stiffens the cornea to prevent further shape changes [6,7].

Scleroderma is a group of rare chronic autoimmune disorders of the connective tissue caused by excessive collagen buildup that results in skin hardening and thickening, followed by scarring [8]. There are two main types of scleroderma: localized scleroderma and systemic sclerosis [8].

Some well-known ocular manifestations in patients with scleroderma are dry eye, skin tightening around the eyelids, impaired glandular function, conjunctival disruptions, cataract changes, and complications of the retina [9,10]. The overall corneal implications of scleroderma were rarely documented in the literature. This could be due to the rare nature of the disorder, which does not particularly affect vision-related functions. As a result, there are limited reports available to assess and document the rare coexistence of both conditions. [11-13].

Managing KC in individuals with autoimmune conditions can pose challenges [14-16]. Our case illustrates that CXL can be a viable and safe intervention for patients with autoimmune conditions. However, it is important to be mindful of potential issues related to impaired healing and active autoimmune disease [14-16].

## Case presentation

**Figure 1:**
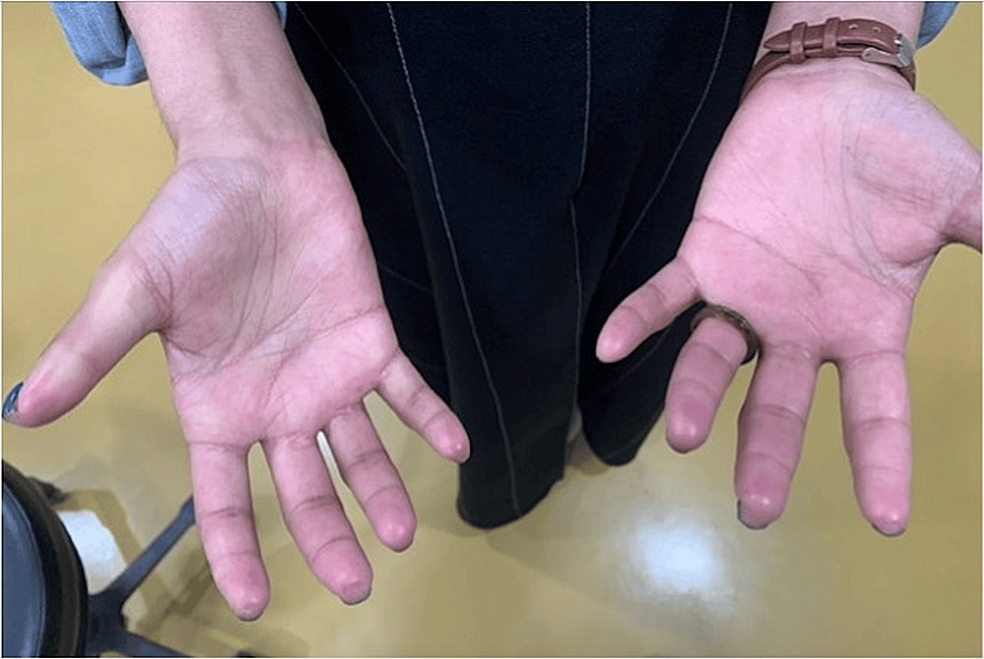
Fused finger, clinical manifestation of scleroderma, that hinders patient from contact lens wear.

**Figure 2:**
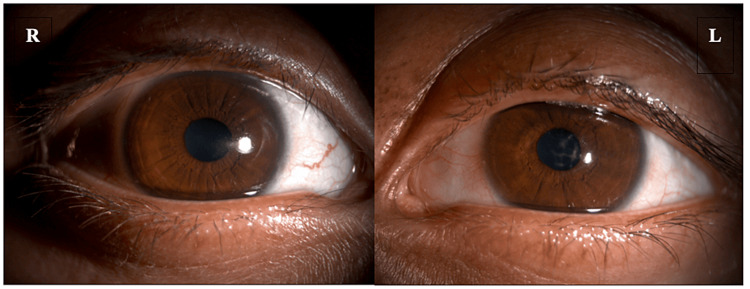
The right eye shows faint scar and left eye a dense paracentral scar.

A 23-year-old lady with a known case of diffuse systemic scleroderma was referred to our tertiary center as a case of KC. She had been complaining of a painless, progressive decrease in vision that was worse in the left eye. She was on prednisolone 5 mg daily and methotrexate 20 mg weekly for her diffuse systemic scleroderma. She had a family history of KC but no history of eye-rubbing. On her first visit, the patient reported that she had been facing difficulties with the contact lenses and required help from others to apply them. It is probably also related to her ventral pterygium (Figure 1). A general inspection of the patient showed a masked face and sclerodactyly. Her hand skin was taut and shiny, with the absence of normal markings. The ocular examination is as follows: uncorrected visual acuity was 20/200 in both eyes. Intraocular pressure was 15 mmHg and 25 mmHg in the right eye and left eye, respectively. Periocular examination revealed thickened, shiny eyelid skin. Anterior segment examination revealed a faint corneal scar and a dense scar with Vogt striae in the right and left eye, respectively (Figure 2). The Schirmer test and tear-break-up time (TBUT) were normal. The conjunctiva was quiet and had a normal appearance. Right eye manifest refraction was −0.5 to 5.25 × 80, with a visual acuity of 20/30. In the left eye, refraction was −10.00 to 8.50 × 170, with no significant improvement in visual acuity (20/200). Corneal tomography showed the right eye to have a steep cornea with a paracentral cone with a mean keratometry of 55.6 D and a K max of 72.5 D. In the left eye, the mean keratometry was 71 D, and the K max was 82.3 D. The thinnest pachymetry was 443 and 338 μm and located inferotemporal in both eyes. The patient had a trial of contact lenses, and her vision reached 20/20 and 20/30 in the right and left eye, respectively. To prevent the further progression of KC in the right eye, the patient underwent uneventful corneal crosslinking in this eye using the Avedro KXL system (Waltham, MS, USA). Benoxinate hydrochloride drops were applied for anesthesia, followed by the application of 99.7% ethyl alcohol to the central cornea for 30 seconds. We performed 9 mm of de-epithelialization using a micro-sponge. The remaining corneal thickness after debridement was 453 μm, as measured by ultrasound contact pachymetry (Tomey SP 3000 pachymeter). A mixture of 0.1% riboflavin in a 20% dextran solution was applied with an induction time of 20 minutes into the cornea until the stroma had absorbed the mixture and stained yellow. The ultraviolet A (UVA) power applied was 15 mW/cm^2^, with an irradiation time of eight minutes. The total treatment time was 16 minutes. At the end of the treatment, the eye was washed with normal saline, and a bandage contact lens was applied. The patient was discharged on prednisolone acetate 1% tapering (QID, TID, BID, and QD, each for one week) and moxifloxacin 0.5% QID for two weeks.

**Figure 3:**
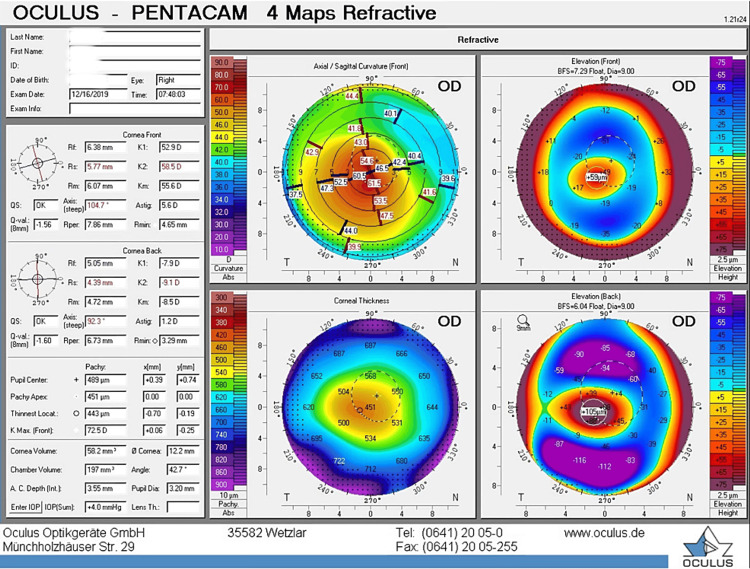
Preoperative in 2019 Pentcam values.

**Figure 4:**
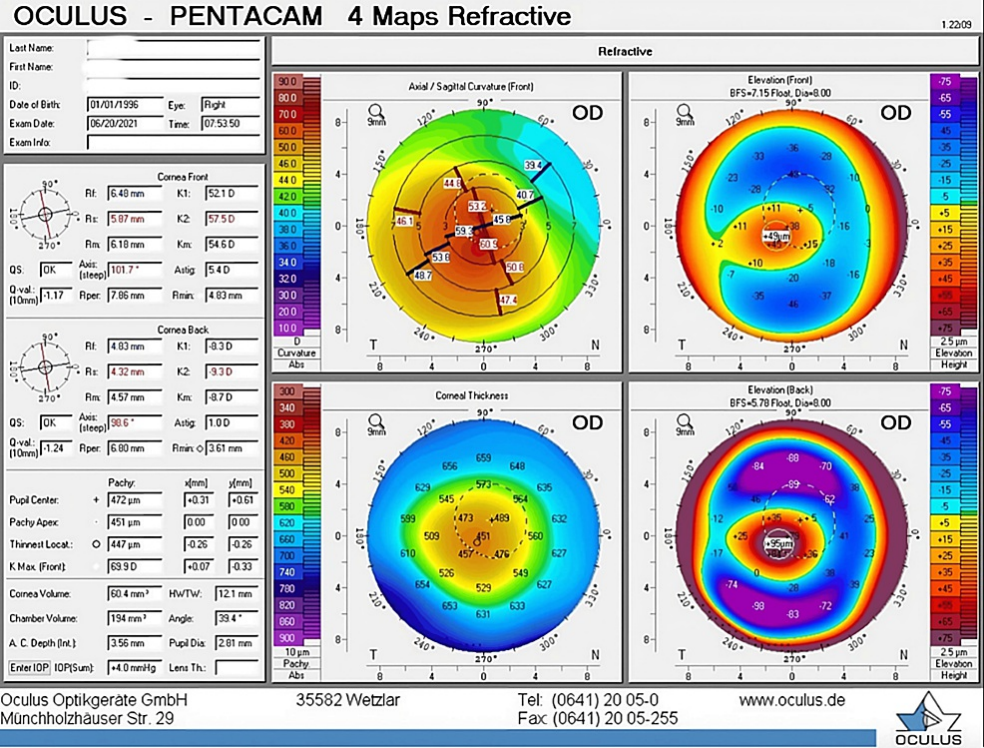
Postoperative Pentcam values.

Followed by a similar tapering course of fluoroemetholone (0.1% for four weeks). Post-op day one, the patient was doing fine with a visual acuity of 20/70. The eye examination revealed a clear cornea and a healing epithelial defect that was otherwise unremarkable. On day 5 post-cxl, the epithelium was healed completely. Further follow-up was unremarkable, with no evidence of epitheliopathy, melting, or unusual corneal haze. Corneal tomography 15 months after treatment showed stabilization of ectasia, with improvement in visual acuity (Figures 3-4).

The patient provided informed consent for the procedures mentioned, indicating a clear understanding and willingness to undergo each intervention separately.

The patient expressed contentment and delight with the results of her treatment during her most recent visit, with no reported complaints or concerns. This positive feedback indicates her overall satisfaction with the care provided and the favorable outcome of the medical intervention, reflecting a successful and gratifying patient experience.

## Discussion

Szucs et al. have studied the ocular manifestations of 51 patients with scleroderma, 102 eyes in total [9,10]. Dry eye disease was the most common ocular disease, followed by eyelid skin changes and cataract development [9,10]. KC remains a very rare association with scleroderma [11,12]. The United States Food and Drug Administration (FDA) has approved CXL for patients with progressive KC and post-laser in situ keratomileusis ectasia [7]. Autoimmune diseases and severe dry eye are some of the relative contraindications for CXL [7].

Alió et al. reported a successful CXL procedure in a patient who had Steven Johnson syndrome. After 15 months, there were no complications, and the cornea was stable [17]. Similarly, Chandratreya et al. also reported successful collagen CXL in a patient with scleroderma and keratoconus. There were no adverse effects, and the cornea remained stable even after six months [13]. These findings are in line with our own results.

However, studies of laser refractive surgery in autoimmune patients have been reported to be successful. In one study, Li et al. studied 42 eyes with collagen vascular disease, including scleroderma, rheumatoid arthritis (RA), systemic lupus erythematosus (SLE), and others [18]. The study included patients with no active ocular disease or history of ocular manifestations. In addition, patients had normal tear film functions and controlled systemic disease. Postoperatively, follow-up examinations revealed the onset of a moderate degree of dry eye syndrome in four eyes (9.5%). There were no instances of corneal haze, melting, flap issues, or interface complications in any of the eyes studied. This outcome aligns with findings by Smith et al., who investigated 49 eyes of 26 patients with autoimmune diseases such as SLE, RA, psoriatic arthritis, scleroderma, inflammatory bowel disease, Graves’ disease, Reiter’s syndrome, and Hashimoto’s disease [15]. In their study, conducted over a mean follow-up of 19 months, none of the patients undergoing laser-assisted in situ keratomileusis (LASIK) experienced corneal thinning, melting, persistent epithelial defects, persistent keratitis, scleral thinning, or scleritis [15]. It is noteworthy that LASIK involves preserving the epithelium, unlike CXL, which may account for the negligible risks of delayed epithelial healing, stromal melt, and infections.

Furthermore, Alkharashi et al. have proposed guidelines for laser refractive surgery in autoimmune disease patients [16]. According to these guidelines, patients with moderate or severe disease and those requiring a multidrug regimen to manage the disease should be excluded. In addition, systemic disease should be under control with no evidence of activity or flare-up for a minimum of six months. Patients should have no history of ocular involvement, and their eye exams, including the Schirmer test and TBUT, should be normal. Our patient had a normal Schirmer test and tear-break-up time on the last follow-up. A rheumatologist and a uveitis specialist should clear the patient for surgery. These guidelines, designed for laser refractive surgery, may find applicability and usefulness in cases involving CXL in patients with autoimmune diseases [15,16].

The literature remains very limited regarding corneal CXL in autoimmune patients [14-16]. This might be due to several factors, including the nature of the disease, medically induced immunosuppression, and the potential risks of delayed epithelial healing, corneal melt, infection, and scarring [14-16]. Future standardized prospective studies are needed to clarify the impact of the disease on the eye and the effect of CXL.

## Conclusions

Keratoconus in patients with autoimmune disease is challenging. Our case has demonstrated that CXL can be an effective and safe intervention for patients with autoimmune conditions. However, concerns about poor healing and active disease need to be considered. Autoimmune disease necessitates rheumatology consultation to control systemic disease before undergoing such intervention.
